# A Core–Shell Au@TiO_2_ and Multi-Walled Carbon Nanotube-Based Sensor for the Electroanalytical Determination of H_2_O_2_ in Human Blood Serum and Saliva

**DOI:** 10.3390/bios12100778

**Published:** 2022-09-20

**Authors:** Ayman Ali Saeed, Mohammed Nooredeen Abbas, Waheed Fathi El-Hawary, Yousry Moustafa Issa, Baljit Singh

**Affiliations:** 1 Applied Organic Chemistry Department, Chemical Industries Research Institute, National Research Centre (NRC), Dokki, Giza 12622, Egypt; 2 Chemistry Department, Faculty of Science, Cairo University, Giza 12613, Egypt; 3 MiCRA Biodiagnostics Technology Gateway & Centre of Applied Science for Health, Technological University Dublin (TU Dublin), D24 FKT9 Dublin 24, Ireland

**Keywords:** electrochemical sensor, core–shell, titanium dioxide, gold nanoparticles, carbon nanotubes, hydrogen peroxide

## Abstract

A hydrogen peroxide (H_2_O_2_) sensor was developed based on core–shell gold@titanium dioxide nanoparticles and multi-walled carbon nanotubes modified glassy carbon electrode (Au@TiO_2_/MWCNTs/GCE). Core–shell Au@TiO_2_ material was prepared and characterized using a scanning electron microscopy and energy dispersive X-ray analysis (SEM/EDX), transmission electron microscopy (TEM), atomic force microscopy (AFM), Raman spectroscopy, X-ray diffraction (XRD) and Zeta-potential analyzer. The proposed sensor (Au@TiO_2_/MWCNTs/GCE) was investigated electrochemically using cyclic voltammetry (CV) and electrochemical impedance spectroscopy (EIS). The analytical performance of the sensor was evaluated towards H_2_O_2_ using differential pulse voltammetry (DPV). The proposed sensor exhibited excellent stability and sensitivity with a linear concentration range from 5 to 200 µM (*R*^2^ = 0.9973) and 200 to 6000 µM (*R*^2^ = 0.9994), and a limit of detection (LOD) of 1.4 µM achieved under physiological pH conditions. The practicality of the proposed sensor was further tested by measuring H_2_O_2_ in human serum and saliva samples. The observed response and recovery results demonstrate its potential for real-world H_2_O_2_ monitoring. Additionally, the proposed sensor and detection strategy can offer potential prospects in electrochemical sensors development, indicative oxidative stress monitoring, clinical diagnostics, general cancer biomarker measurements, paper bleaching, etc.

## 1. Introduction

Carbon nanomaterials, including single-walled carbon nanotubes (SWCNTs), multi-walled carbon nanotubes (MWCNTs), nanofibers, activated carbon and graphene, have unique properties such as chemical stability and durability, high electrical conductivity, mechanical strength and a high surface-to-volume ratio which collectively makes them an excellent choice for electrochemical sensors and biosensors development [1,2,3,4,5,6,7,8,9,10]. Carbon nanotubes (CNTs) are an excellent and promising option in fabricating electrochemical sensors owing to their remarkable electrical and thermal conductivity, high surface area, chemical stability and mechanical properties [11,12,13,14]. Metal nanoparticles, due to their excellent conductivity, surface area and remarkable electrocatalytic properties, are considered ideal candidates for the electrochemical detection of hydrogen peroxide (H_2_O_2_). They are capable of promoting electron transfer processes and offer abundant catalytic active sites during the H_2_O_2_ redox reaction. Carbon-based nanomaterials can be mixed with metal nanoparticles to create composites that have excellent synergistic features, which can improve the sensitivity and overall performance of the modified electrodes.

Core–shell nanomaterials (CSNs) are composite materials with a core–shell structure formed by an inner layer of one material (core) and an exterior layer of another material (shell) [15]. The shell materials are frequently chosen based on their nature and the targeted application. Many advantages can be achieved by carefully selecting shell material, including improved optical/electrical/magnetic properties, multifunctional capability, thermal stability or dispersibility of the materials, reduced precious material content and efficient use. In the core–shell structure, the size and kind of core are also crucial factors. The main advantage of CSNs is that the distinct properties of the core and shell can be combined in a single material, resulting in improved electrocatalytic activity and new physical and chemical properties that are inaccessible or unavailable from the individual components due to lack of this synergistic effect. In order to maintain the nanoparticles’ stability and chemical activity, the core material is also safeguarded against migration and aggregation. CSNs have been studied extensively for a variety of biological applications, including drug administration, cancer treatment, bioimaging, cell labelling, genetic engineering, methanol electrooxidation and so on [16,17]. Due to their higher surface area, superior catalytic activities and biocompatibility, CSNs have been exploited as signal amplifiers and considered promising electron modifiers to create novel sensing platforms, including electrochemical sensors and biosensors. The excellent features of CSNs make them ideal candidates for developing sensitive and novel electrochemical sensors [18,19,20,21,22,23,24,25,26].

Accurate, sensitive and reliable detection of H_2_O_2_ has received substantial attention in analytical applications due to the importance of H_2_O_2_ in various areas. Hydrogen peroxide is a common peroxide that is used in biological systems, medical diagnosis, environmental analysis, food and many other applications [27,28,29]. Furthermore, H_2_O_2_ as a member of reactive oxygen species (ROS), is considered one of the crucial oxidative stress biomarkers. The excessive level of H_2_O_2_ can cause a series of diseases such as Alzheimer’s, Parkinson’s, myocardial infarction, inflammatory lung diseases, cancer, etc. [30,31]. Therefore, it is essential to develop an effective method for rapid and reliable monitoring of H_2_O_2_ [32,33].

Several analytical methods have been developed for H_2_O_2_ determination, including spectrophotometry [34,35], chemiluminescence [36,37,38], fluorescence [39,40,41], chromatography [42,43] and electrochemical sensors [44,45,46,47,48,49,50,51,52,53,54,55,56,57,58,59,60,61,62,63,64,65]. However, due to the technical drawbacks of the traditional methods (low sensitivity and selectivity, laborious, time-consuming and complicated instrumentation), electrochemical sensors have received more attention due to their associated practical advantages, including high sensitivity, portability, simplicity, cost-effectiveness, rapid response time and ease of fabrication and operation. Electrochemical sensors for H_2_O_2_ determination are mainly based on enzymatic and non-enzymatic approaches, but due to drawbacks of enzymatic sensors such as instability (temperature, pH-related challenges), shelf-life and immobilization procedures, non-enzymatic sensors have received greater attention [44,45,46,47,48,49,50,51,52,53,54,55,56,57,58,59,60,61].

In this paper, we describe the fabrication of a hydrogen peroxide sensor based on modifying the surface of a glassy carbon electrode (GCE) with a gold@titanium dioxide (Au@TiO_2_) core–shell nanoparticle and multi-walled carbon nanotubes. The characterization of the core–shell material and fabricated electrode was investigated thoroughly, and the electroanalytical performance of the sensor was studied and discussed in detail. The proposed sensor exhibited excellent electroanalytical performance and electrocatalytic activity toward H_2_O_2_ reduction. The practicality of the proposed sensor was tested by measuring H_2_O_2_ in human serum and saliva samples which demonstrates its potential for H_2_O_2_ monitoring in real-world samples. Additionally, the proposed sensor and detection strategy may offer potential prospects in electrocatalysts and electrochemical sensors development, as well as in other applications, including indicative oxidative stress monitoring, clinical diagnostics, general cancer biomarker measurements, food processing, paper bleaching and environmental analysis.

## 2. Materials and Methods

### 2.1. Materials

Gold(III) chloride hydrate (HAuCl_4_.H_2_O), trisodium citrate and multi-walled carbon nanotubes (MWCNTs) were purchased from Sigma-Aldrich. Titanium(IV) tetraisopropoxide (TTIP, 98%) and sodium dodecyl sulphate (SDS, 85%) were purchased from ACROS ORGANICS. H_2_O_2_ (30%) was purchased from Advent Chembio Pvt. Ltd. Potassium ferricyanide was purchased from VEB Laborchemie Apolda, while potassium ferrocyanide was purchased from BDH.

### 2.2. Apparatus and Measurements

The cyclic voltammetry (CV), electrochemical impedance spectroscopy (EIS) and differential pulse voltammetry (DPV) measurements were performed using CH Instruments Inc. (CHI 660D). A glassy carbon electrode (GCE, 3 mm diameter) was used as a working electrode, while a platinum wire and an Ag/AgCl reference electrode (3 M KCl) were used as counter and reference electrodes, respectively. The experiments of electrochemical characterization were carried out in 5 mM [Fe(CN)_6_]^3−/4−^ solution as a redox probe from +0.7 V to −0.4 V, while the different concentrations of H_2_O_2_ (5 to 6000 µM) were prepared in 0.1 M PBS saturated with N_2_.

Raman spectroscopy was carried out using WITec alpha300 R at 532 nm. Zeta potential was measured using Malvern Zetasizer zs. Transmission electron microscopy (TEM) images were recorded using a JEOL JEM-1230. Surface morphology characterization and identification were performed using scanning electron microscopy (SEM, TESCAN VEGA3) coupled with energy dispersive X-ray analysis (EDX, BRUKER) and an atomic force microscope (5600LS, Agilent, Santa Clara, CA, USA). X-ray diffraction (XRD) was performed using an X-ray diffractometer (BRUKER, D8 DISCOVER).

### 2.3. Synthesis of Gold Nanoparticles (AuNPs)

Gold(III) chloride solution (147 mM, 100 µL) was added to distilled water (50 mL) to prepare 0.01% (*w/v*) solution and heated until boiling. In total, 1% (*w/v*, 2 mL) trisodium citrate was added under vigorous stirring, and the color of the solution changed from pale yellow to blue and finally red. The solution was cooled to room temperature and stored at 4 °C. The final concentration of the as-prepared gold nanoparticles was 58 ppm.

### 2.4. Synthesis of Core–Shell AuNPs@TiO_2_ and AuNPs@TiO_2_/MWCNTs

Au@TiO_2_ was prepared by modifying the preparation methods mentioned in previous reports [66,67,68]. Au@TiO_2_ with different wt% of core Au (1.09, 2.17, 4.24, 8.14 and 11.74%) was prepared. For Au@TiO_2_ (8.14% Au), 20 mL of the prepared AuNPs stock solution was added dropwise to 20 mL of 0.1 mol/L SDS solution under vigorous stirring for 15 min. The mixture was centrifuged at 12,000 rpm for 10 min. The SDS-capped AuNPs were settled at the bottom of the centrifuge tube while the supernatant was removed. The SDS-capped AuNPs were washed three times with distilled water to remove the excess free SDS. In total, 50 µL of TTIP were mixed with 500 µL of isopropanol, and the mixture was added dropwise to the washed SDS-capped AuNPs under vigorous stirring. The formed precipitate was washed with ethanol and allowed to dry. The dried product was calcinated at 500 °C for 4 hrs. Then, 1 mg of AuNPs@TiO_2_ was suspended in 980 µL distilled water, and 20 µL of 1 mg/mL MWCNTs were added. Finally, the mixture was sonicated for 30 min.

### 2.5. Fabrication of H_2_O_2_ Electrode (Au@TiO_2_/MWCNTs/GCE) 

Before making the electrochemical measurements, glassy carbon working electrodes were polished using alumina powders (1.0, 0.3 and 0.05 µm size), followed by washing with acetone and finally with deionized water and dried at room temperature. The glassy carbon electrode was firstly activated by recording CV in 0.5 M H_2_SO_4_ from 1.4 to −0.2 V for 20 cycles at a scan rate of 0.1 V/s. The electrode was then washed with distilled water and allowed to dry. Then, 5 µL of the Au@TiO_2_/MWCNTs were drop-casted onto the electrode surface and allowed to dry at room temperature.

### 2.6. Real Sample Analysis

The proposed H_2_O_2_ sensor (Au@TiO_2_/MWCNTs/GCE) was tested for the determination of H_2_O_2_ in human serum and saliva samples. Three volunteers participated in this study, two males and one female, with different ages varying from 24 to 40 years. Saliva samples were collected in the morning, volunteers were asked to rinse their mouths with water for 1 min, and then samples were collected after 3 min. Collected saliva samples were centrifuged for three minutes at 4000 rpm in order to settle down any food residues, and clear saliva was diluted 10 times with 0.1 M PBS (pH 7.4) for analysis. For the serum analysis, a 10 mL sample was collected, and the blood was allowed to coagulate for 1 h, then centrifuged at 5000 rpm for 5 min, and finally, the upper serum layer was collected and diluted 20 times with PBS for analysis.

## 3. Results and Discussion

### 3.1. UV-Visible Spectroscopy

**Figure 1:**
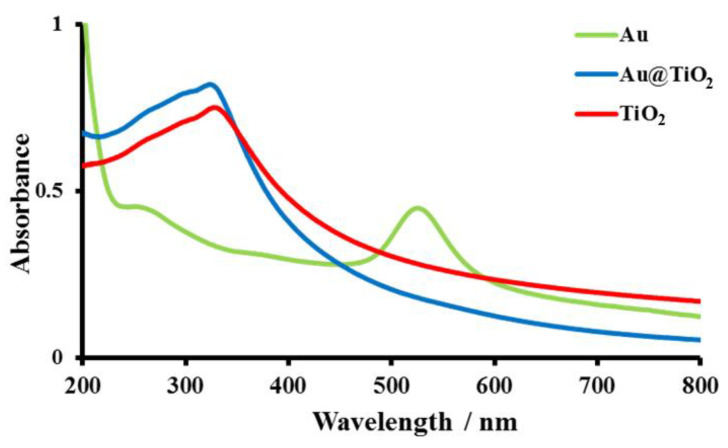
UV-Visible absorption spectra of AuNPs, TiO_2_ and Au@TiO_2_ materials.

In order to ensure the success of Au@TiO_2_ preparation, UV-Visible absorption spectra were recorded for AuNPs, TiO_2_ and Au@TiO_2_. As shown in Figure 1, in the case of AuNPs, the spectrum showed the characteristic absorption peak at 524 nm. TiO_2_ nanoparticles are commonly used as an additive in most sunscreen formulations due to their broad absorption within the UVB and UVA region, with a maximum of 327.5 nm. The successful preparation of Au@TiO_2_ was accompanied and confirmed by the disappearance of the AuNPs peak, which is attributed to the complete coverage of the core (Au) by the TiO_2_ shell. Moreover, a slight blue shift was observed from 327.5 nm to 323.5 nm.

### 3.2. Raman Spectroscopy

**Figure 2:**
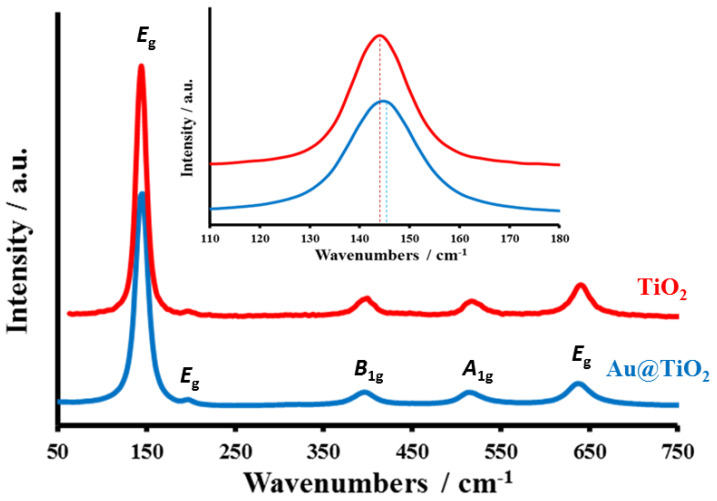
Raman spectra of the prepared TiO_2_ and Au@TiO_2_ (Au is 8.14%) materials.

The TiO_2_ and Au@TiO_2_ materials were characterized by Raman spectroscopy, and the associated spectra are shown in Figure 2. The existence of bands at 144 (*E*_g_), 198 (*E*_g_), 396 (*B*_1g_), 515 (*A*_1g_) and 638 (*E*_g_) cm^−1^ are unquestionably linked to the TiO_2_ anatase phase [69,70,71]. In the case of Au@TiO_2_, the typical *E*_g_ peak of TiO_2_ at 144 cm^−1^ shifted to a higher wavenumber (146 cm^−1^), indicating more crystalline disorders in the anatase TiO_2_. These crystalline disorders, which developed at the point where Au and TiO_2_ came into contact, impact the vibrational frequency of anatase TiO_2_ and serve as traps for the produced photoelectrons.

### 3.3. X-ray Diffraction (XRD)

**Figure 3:**
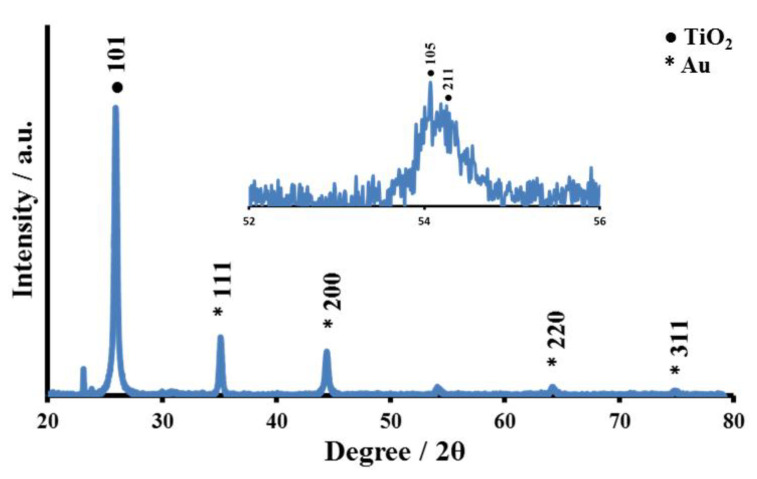
X-ray diffraction pattern for the prepared core–shell Au@TiO_2_ material (Au = 8.14%).

The crystalline nature of the core–shell Au@TiO_2_ material was characterized by X-ray diffraction (XRD), as shown in Figure 3. The samples show two series of peaks, which can be attributed to the anatase-TiO_2_ and the face-centered-cubic Au. Planes (101), (200), (105) and (211) of anatase-TiO_2_ are responsible for the peaks at 2*θ* of 25.7°, 44.4°, 54.0° and 54.3°, while planes (111), (200), (220) and (311) of face-centered-cubic Au are associated with the remaining peaks at 2*θ* of 38.2°, 44.4°, 64.7° and 77.7°, in agreement with the literature [72].

### 3.4. Zeta-Potential Analyzer and Size Distribution

**Figure 4:**
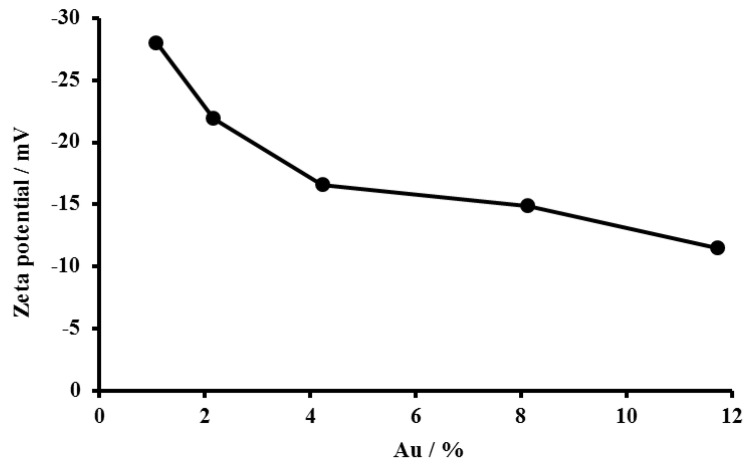
Zeta potential of the prepared core−shell Au@TiO_2_ material with varying Au content (1.09, 2.17, 4.24, 8.14 and 11.74%).

Higher zeta potential values indicate that the periphery surface charge of the nanoparticles is higher, which encourages repulsion and prevents the formation of aggregates, a sign of the stability of the core–shell nanoparticles. Au@TiO_2_ core–shell with different wt% of Au (1.09, 2.17, 4.24, 8.14 and 11.74%) were prepared, and their Zeta potentials were measured to evaluate the effect of the weight percent of the core gold on the surface charge and stability of the whole core–shell material. As seen in Figure 4, the increase in the weight percent of core gold is accompanied by a positive increase in the value of Zeta potential until it reaches nearly stable values between 4.24% and 8.14%. Therefore, we decided to use 8.14% of Au content in further studies.

### 3.5. Transmission Electron Microscopy (TEM)

**Figure 5:**
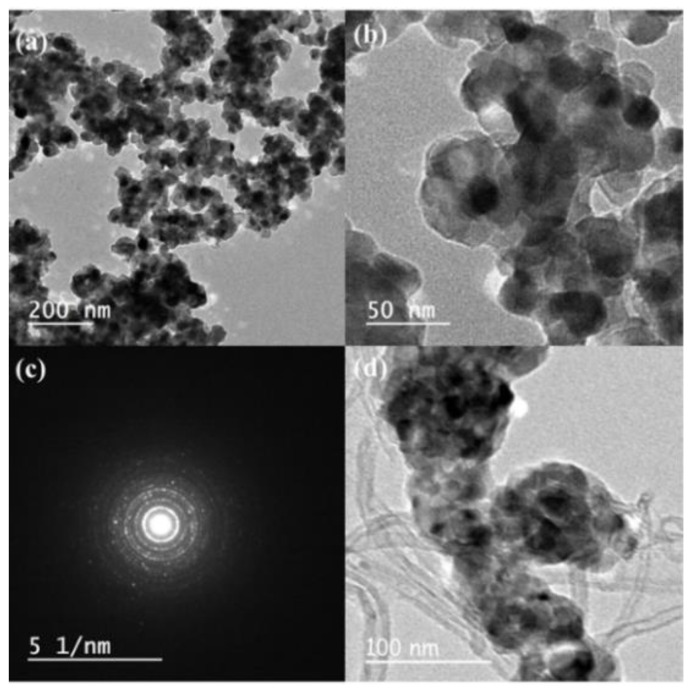
TEM images of (**a**,**b**) Au@TiO_2_ with 8.14% Au; (**c**) selected area electron diffraction pattern; (**d**) TEM image of Au@TiO_2_/MWCNTs composite material.

TEM analysis was performed to directly measure the size, size distribution and morphology of Au@TiO_2_ and Au@TiO_2_/MWCNTs, which are shown in Figure 5a–d. The spherical gold nanoparticles were observed as a dark core, with about 13–15 nm diameter, in the center and totally encapsulated by a brighter shell of TiO_2_. It can be observed that gold nanoparticles are well-dispersed and uniformly incorporated in the TiO_2_ matrix without any significant agglomeration. Figure 5c shows the selected area electron diffraction pattern, which agrees with the XRD data and reveals that the compositions of core–shell (Au and TiO_2_) reflect their signatures of crystal planes in the hybrid or composite form. The combination of MWCNTs with core–shell Au@TiO_2_ is confirmed and presented in Figure 5d. 

### 3.6. Scanning Electron Microscopy and Energy Dispersive X-ray Analysis (SEM and EDX)

**Figure 6:**
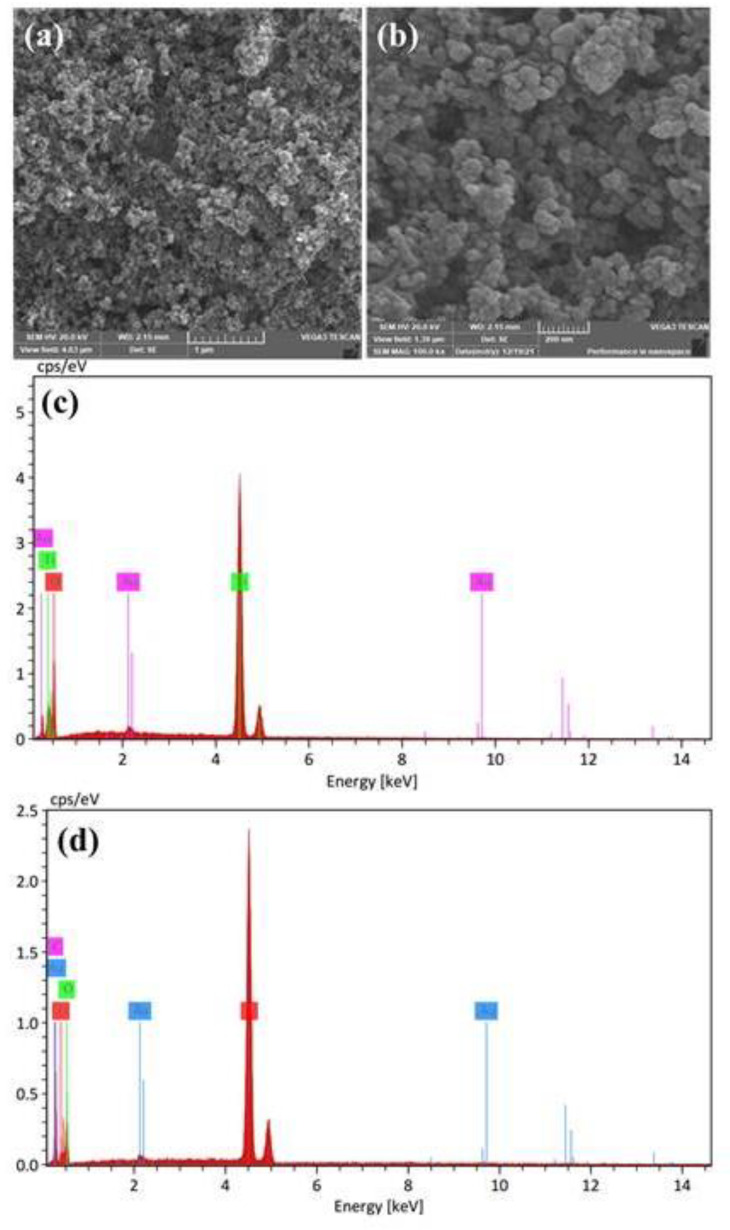
SEM images of Au@TiO_2_ material (Au = 8.14%) at different magnifications; (**a**) 30 kx and (**b**) 100 kx. (**c**,**d**) EDX profiles for Au@TiO_2_ and Au@TiO_2_/MWCNT materials confirming the presence of elements as expected.

SEM images were recorded in order to describe the surface morphology and distribution of the Au@TiO_2_ material. As shown in Figure 6a,b, the presence of spherical and uniformly distributed particles was observed with a size of approximately 30.29 nm (diameter). The modification of the electrode surface with such core–shell material promises a high surface area and better electrocatalytic activity. The elemental analysis for Au@TiO_2_ and Au@TiO_2_/MWCNT materials was performed, and EDX profiles are shown in Figure 6c,d. The EDX profile for Au@TiO_2_/MWCNT (Figure 6d) confirms the presence of Ti, O and Au and C with a weight percent of 39.88%, 46.92%, 7.76% and 5.45%, respectively. The Au amount observed from the EDX measurements is in approximate agreement with the theoretical value of 8.14%.

### 3.7. Atomic Force Microscopy (AFM)

**Figure 7:**
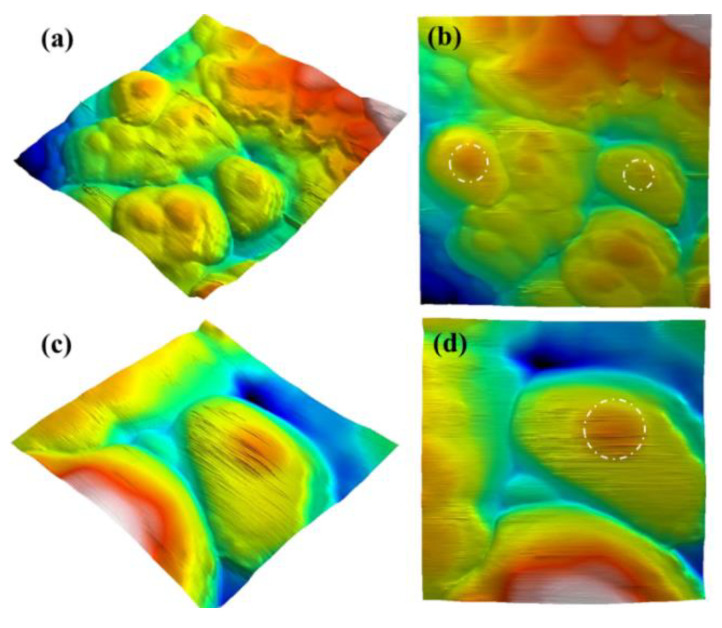
AFM images of the core–shell Au@TiO_2_ material; (**a**,**c**) 3D images and (**b**,**d**) plane images.

AFM analysis was performed to characterize the surface morphology of the Au@TiO_2_-modified surface, and the images are shown in Figure 7**.** It is obvious that the topography of the core–shell-modified surface has a high surface roughness with a root mean square height (Sq) of 35.4 nm. As shown in Figure 7a,c, the 3D images confirm the success of the core–shell preparation process. Protrusions were observed in the TiO_2_ shell as the embedded gold nanoparticles in the core push the TiO_2_ shell out. 

### 3.8. Cyclic Voltammetry 

**Figure 8:**
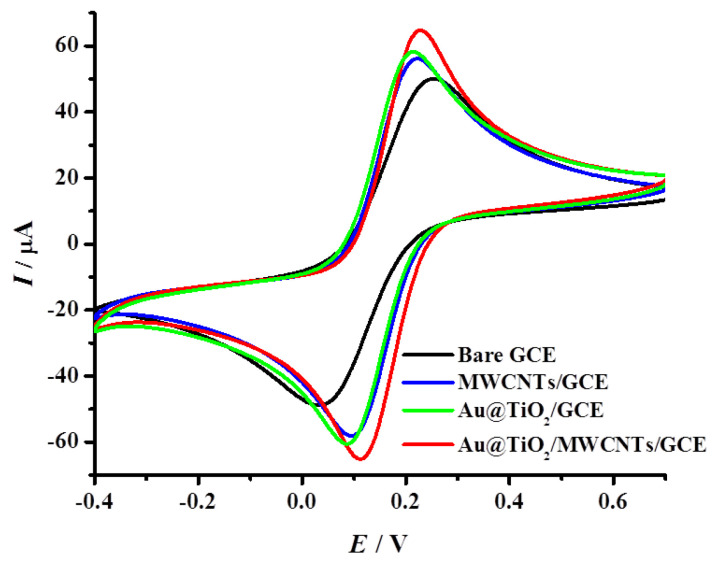
Cyclic voltammetric responses for bare GCE, MWCNTs/GCE, Au@TiO_2_/GCE and Au@TiO_2_/MWCNTs/GCE in 5 mM [Fe(CN)_6_]^3−/4−^/0.1 M KCl at a scan rate of 100 mV/s.

Cyclic voltammetry characterization (Figure 8 and Table 1) was performed by recording the voltammograms for various stages of electrode modification in 5 mM [Fe(CN)_6_]^3−/4−^ (1:1) in 0.1 M KCl at a scan rate of 100 mV/s (potential window 0.7 to −0.4 V). The current response for the MWCNTs modified electrode (*I*_Oxi_ = 60.71 µA and *I*_Red_ = −61.61 µA, Δ*E* = 129 mV) was improved very significantly compared to the bare GCE (*I*_Oxi_ = 45.83 µA, *I*_Red_ = −44.61 µA, Δ*E* = 220 mV). This is attributed to the high conductivity of MWCNTs, which improve the electron transfer kinetics between the electrode surface and redox couple. 

The drop-casting of the as-prepared core–shell Au@TiO_2_ material onto GCE also showed an increase in the current response (*I*_Oxi_ = 61.81 µA, *I*_Red_ = −63.04 µA) with a decrease in Δ*E* value (126 mV) and this is attributed to the combined catalytic activity of TiO_2_ and Au. Au@TiO_2_/MWCNTs/GCE showed a much better current response compared to bare GCE, MWCNTs/GCE and Au@TiO_2_/GCE with *I*_Oxi_ = 69.77 µA, *I*_Red_ = −69.56 µA and the lowest Δ*E* (115 mV) was observed. This improvement is attributed to the synergistic effect of the core–shell Au@TiO_2_ structure and the high electrical conductivity of MWCNTs.

### 3.9. Electrochemical Impedance Spectroscopy

**Figure 9:**
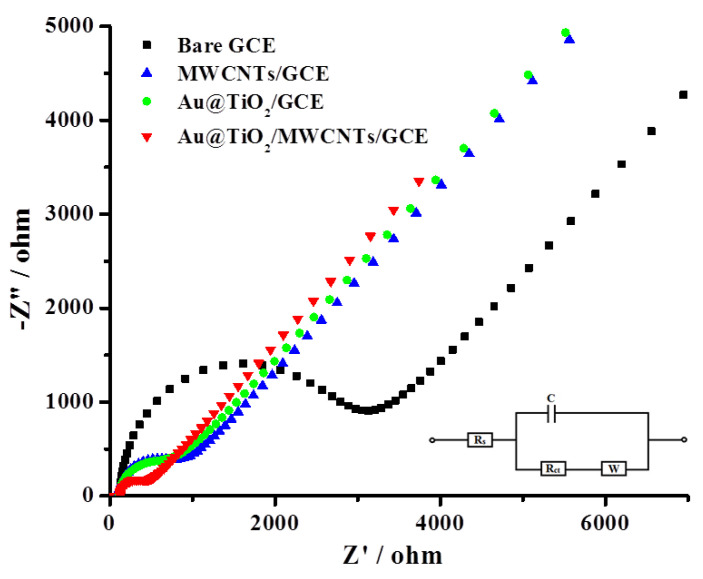
Nyquist plot for modified (labeled) and bare glassy carbon electrodes in 5 mM [Fe(CN)_6_]^3−/4^/0.1 M KCl. Insert shows the Randel circuit used for data fitting.

EIS analysis was performed and recorded in 5 mM [Fe(CN)_6_]^3−/4−^ solution at 0.22 V with a frequency range from 0.1 Hz to 10^5^ Hz (Figure 9 and Table 1) to support the cyclic voltammetry results and to confirm the *R*_ct_ values which agree well with the voltammetry findings. The bare GCE showed an *R*_ct_ of 2610 Ω, which was decreased very significantly (634 Ω) after modifying the GCE surface with MWCNTs. This is due to the reason that the electron process kinetics is faster due to the high electrical conductivity of MWCNTs, which ultimately facilitates the electron transfer and decreases the resistance. Au@TiO_2_-modified GCE also showed a decrease in the *R*_ct_ value (529 Ω), but the proposed electrode material Au@TiO_2_/MWCNTs/GCE exhibited the best and lowest charge transfer resistance (281 Ω) among all the tested materials and is attributed to the synergistic effect of the core–shell Au@TiO_2_ nanostructure and high conductivity of MWCNTs.

### 3.10. Analytical Performance (H_2_O_2_ Sensing)

**Figure 10:**
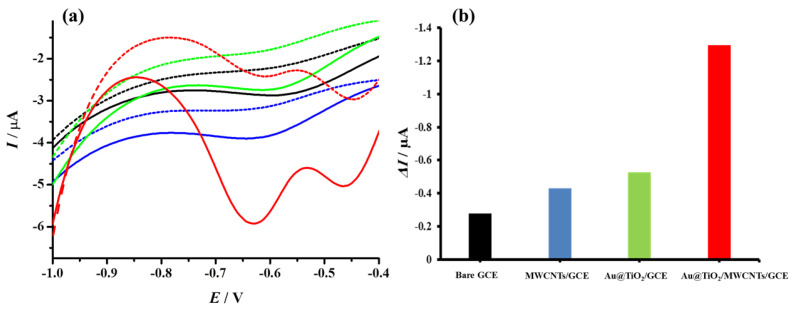
(**a**) Differential pulse voltammograms recorded in PBS (dashed lines) and 300 µM H_2_O_2_ (solid lines) for various stages of electrode modification, Bare GCE (black); MWCNTs/GCE (blue); Au@TiO_2_/GCE (green); Au@TiO_2_/MWCNTs/GCE (red). (**b**) Histogram represents the corresponding change in current values (Δ*I*).

In order to confirm the synergistic effect on H_2_O_2_ sensing, DPVs were recorded in N_2_-saturated PBS for the different stages of electrode modification. As shown in Figure 10, the increase in the current response was 1.5 and 1.9 times in the case of MWCNTs/GCE and Au@TiO_2_/GCE, respectively, while an increase of 4.6 times was observed in the case of Au@TiO_2_/MWCNTs/GCE compared to the bare glassy carbon electrode.

**Figure 11:**
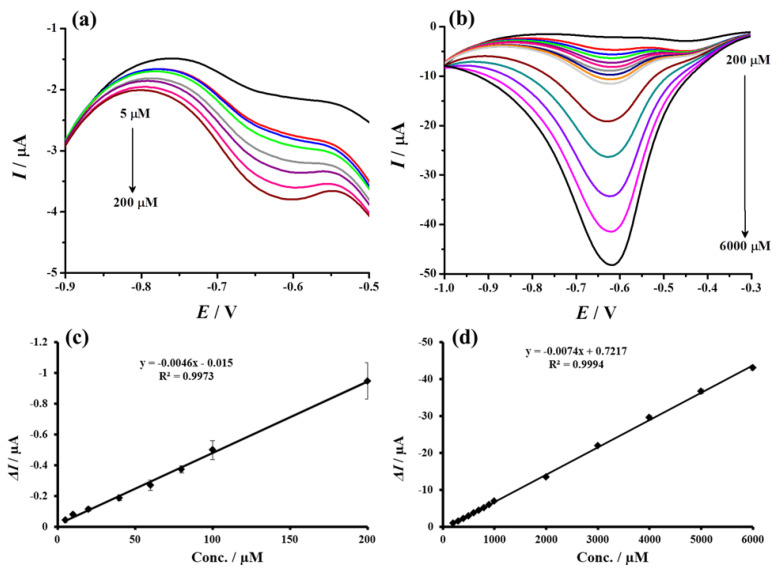
Differential pulse voltammograms recorded using proposed sensor (Au@TiO_2_/MWCNTs/GCE towards different concentrations of H_2_O_2_ (**a**) 5 to 200 µM and (**b**) 200 to 6000 µM) in N_2_-saturated PBS (pH 7.4)). (**c**,**d**) Corresponding calibration curves from the current responses vs. H_2_O_2_ concentrations plot.

The DPV response of the proposed electrode (Au@TiO_2_/MWCNTs/GCE) towards different H_2_O_2_ concentrations (PBS, pH 7.4) was recorded and shown in Figure 11. The reduction current of the hydrogen peroxide (at −0.62 V) increased with H_2_O_2_ concentration and showed a linear response from 5 to 200 µM (y = −0.0046x − 0.015) and from 200 to 6000 µM (y = −0.0074x + 0.7217) with coefficient of determination (*R*^2^) of 0.9973 and 0.9994, respectively. The calculated limit of detection (LOD) was 1.4 µM based on 3σ calculations. The analytical performance of the proposed sensor was compared to other relevant and reported results and is summarized in Table 2. It is clearly evident that the proposed sensor exhibited an excellent response in terms of linear range and detection limit.

#### 3.10.1. Stability and Reproducibility of H_2_O_2_ Sensor

**Figure 12:**
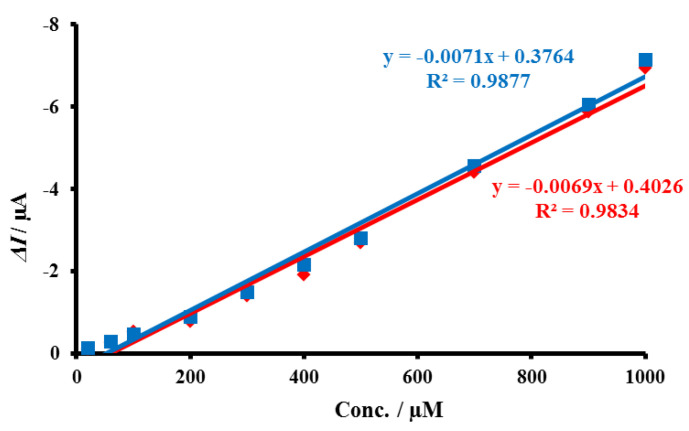
Stability study for the determination of H_2_O_2_ (20–1000 µM) after 50 days (red line) using the proposed sensor (Au@TiO_2_/MWCNTs/GCE) compared to the response observed initially (blue line).

Stability is an important parameter to describe the performance of an electrochemical sensor. The stability of the Au@TiO_2_/MWCNTs/GCE sensor was investigated by measuring the current responses for different concentrations of H_2_O_2_ (20–1000 µM, Figure 12) after 50 days and compared with the initial response recorded. The current response for H_2_O_2_ after 50 days was found to be 97.1% of its initial response, and this reveals the acceptable stability and sufficient lifetime of the proposed sensor. The reproducibility of three freshly prepared Au@TiO_2_/MWCNTs/GCEs was tested by measuring the current responses of 200 µM H_2_O_2_, and the calculated %RSD was 4.63%.

#### 3.10.2. Selectivity of H_2_O_2_ Sensor

Possible interferences which may occur during the determination of H_2_O_2_ in the biological samples were examined using the proposed sensor (Au@TiO_2_/MWCNTs/GCE). The interference study was performed by measuring the current response of 200 µM H_2_O_2_, and then the change in the current response was recorded in the presence of the common interferents (ascorbic acid, glucose, methionine, cysteine and uric acid) with 10-fold concentration (2 mM) compared to H_2_O_2_. As shown in Table 3, the effect of these interferents on H_2_O_2_ determination is expressed as a percentage recovery from the initial H_2_O_2_ current response (before the interferents were added). Even with such high concentrations of interferents (10-fold), the results reveal that the proposed sensor is quite selective towards H_2_O_2_.

### 3.11. Real Sample Analysis

The diluted real samples (human saliva and serum samples) were considered, and analysis was performed in triplicate for different concentrations of H_2_O_2_ by the standard addition method. The recovery values of the proposed sensor are shown in Table 4, which vary from 85.3 to 117.9%. The obtained results reveal that the proposed sensor exhibits a reliable response and excellent practicality for the electroanalytical determination of H_2_O_2_ in real biological samples.

## 4. Conclusions

An electrochemical non-enzymatic biosensor based on Au@TiO_2_ core–shell nanoparticles and muti-walled carbon nanotubes was developed and investigated for H_2_O_2_ determination. The success of the Au@TiO_2_ preparation process was confirmed and characterized by various techniques. The different electrode modification stages were electrochemically characterized using cyclic voltammetry and electrochemical impedance spectroscopy in 5 mM [Fe(CN)_6_]^3−/4−^ in order to confirm the synergistic effect of Au@TiO_2_ and MWCNTs. The synergistic effect was further confirmed during differential pulse voltammetry analysis of H_2_O_2_ (300 µM) in 0.1 M N_2_-saturated PBS (pH 7.4). The analytical performance of the modified electrode (Au@TiO_2_/MWCNTs/GCE) was investigated by plotting the calibration curves of different concentrations of H_2_O_2_ varying from 5 to 6000 µM. The results showed that by increasing the H_2_O_2_ concentration, the reduction current of the hydrogen peroxide at −0.62 V increases, and the current values showed a linear response from 5 to 200 µM (*R*^2^ = 0.9973) and 200 to 6000 µM (*R*^2^ = 0.9994). The calculated limit of detection was 1.4 µM and attributed to the synergistic effect of Au@TiO_2_ and MWCNTs.

The proposed sensor exhibited good selectivity over the possible interferents (ascorbic acid, glucose, methionine, cysteine and uric acid at a 10-fold concentration level compared to H_2_O_2_). The lifetime of the sensor/electrode reached 50 days with a decrease of only 2.9% of the original response (current). The developed sensor was further tested for the determination of H_2_O_2_ in real biological samples (human serum and saliva) using the standard addition method. The observed response and recovery results support the potential of the proposed sensor for H_2_O_2_ monitoring in future real-world sample analysis.

## Figures and Tables

**Table 1:** CV and EIS performance data for modified and bare electrodes in [Fe(CN)_6_]^3−/4−^.

Electrode	*I_Oxi_* (µA)	*E* (mV)	*I_Red_* (µA)	*E* (mV)	Δ*E* (mV)	*R*_s_ (Ω)	*R*_ct_ (Ω)	*C* (µF)	*W* (mΩ)
**Bare GCE**	45.83	252	−44.61	32	220	128.4	2610	0.49	0.143
**MWCNTs/GCE**	60.71	223	−61.61	94	129	113.9	634	0.91	0.167
**Au@TiO_2_/GCE**	61.81	213	−63.04	87	126	118.4	529	1.13	0.149
**Au@TiO_2_/MWCNTs/GCE**	69.77	228	−69.56	113	115	124.8	281	1.00	0.266

**Table 2:** Comparison of the proposed sensor with the recently reported H_2_O_2_ sensors.

Electrode Material	Linear Range	LOD (µM)	Ref.
GO-Fe_3_O_4_-PAMAM-Pd/GCE	0.05–160 μM	0.01	[48]
Pd/TNM@rGO	up to 12 mM	0.0025	[49]
GQDs-CS/MB/GCE	1.0 µM–2.9 mM 2.9–11.78 mM	0.7	[50]
Paper/CNTs/AgNPs	1 μM–700 μM	-	[51]
CuNPs-rGO	up to 18 mM	601	[52]
LSG-Ag	0.1–10 mM	7.9	[53]
α-MoO_3_/GO/GCE	0.92 μM–2.46 mM	0.31	[54]
PtNPs@SPCEs	0–215 µM	1.9	[55]
MPS electrode	10 and 5000 μM	4.35	[56]
Pt-Pd/CFME	5–3920 µM	0.42	[57]
NiCoSe_2_/GCE	0.05 to 402 µM	0.03	[58]
Cu@Pt/C	0.50 μM–32.56 mM	0.15	[59]
Fe_3_O_4_@PEI@AuNPs-GRE	0.2–500.0 μM	0.07	[60]
Cu/Cu_2_O/FTO	0.2–2000 μM	0.04	[61]
AuNPs/*n*-GaN	40 µM–1 mM	10	[62]
Pd/AuNPs	0.5–6 mM	-	[63]
Ni−Bi/CC	0.1 μM–0.5 mM	0.00085	[64]
PtNP/rGO–CNT/PtNP/SPCE	25–1000 µM	4.3	[65]
Au@TiO_2_/MWCNTs/GCE	5–200 µM and 200 µM–6 mM	1.4	This work

PAMAM: Poly(amidoamine) dendrimer; TNM: *tert*-Nonyl mercaptan; GQDs: Graphene quantum dots; CS: Chitosan; MB: Methylene blue; LSG; Laser scribed graphene; SPCEs: Screen-printed carbon electrodes; MPS: Macroporous Silicon; CFME: Carbon fiber microelectrode; PEI: Polyethyleneimine; GRE: Graphite rod electrode; FTO: Fluorine doped tin oxide; CC: Carbon cloth.

**Table 3:** The H_2_O_2_ (200 µM) recovery data for the proposed electrode in the presence of common interferents (10-fold concentration, 2 mM).

Interferent (10-Folds)	Recovery (%) (*n* = 3)
Ascorbic acid	89.11 ± 0.50
Glucose	102.45 ± 0.87
Methionine	96.21 ± 1.23
Cysteine	105.65 ± 0.68
Uric acid	97.31 ± 0.73

**Table 4:** Application of the proposed sensor Au@TiO_2_/MWCNTs/GCE for real sample analysis (human serum and saliva samples).

Sample	Added (µM)	Found in Serum (µM) (*n* = 3)	Recovery (%)	Found in Saliva (µM) (*n* = 3)	Recovery (%)
1	20	18.02 ± 0.94	90.09	18.33 ± 0.71	91.63
	40	38.10 ± 0.89	95.24	38.28 ± 0.60	95.71
	60	51.17 ± 0.70	85.29	70.74 ± 1.43	117.90
2	20	20.70 ± 1.20	103.48	21.30 ± 0.87	106.52
	40	37.41 ± 0.64	93.53	43.98 ± 0.69	109.95
	60	57.39 ± 0.56	95.65	63.85 ± 0.45	106.41
3	20	22.11 ± 0.88	110.54	22.76 ± 1.02	113.80
	40	38.10 ± 1.09	95.24	39.63 ± 0.70	99.08
	60	51.17 ± 0.79	85.29	68.30 ± 0.83	113.84

## Data Availability

The data presented in this study are available on request from the corresponding author.
